# Recovery of Waste Polyurethane from E-Waste. Part II. Investigation of the Adsorption Potential for Wastewater Treatment

**DOI:** 10.3390/ma14247587

**Published:** 2021-12-10

**Authors:** Vincenzo Santucci, Silvia Fiore

**Affiliations:** Department of Engineering for Environment, Land, and Infrastructures (DIATI), Politecnico di Torino, Corso Duca degli Abruzzi 24, 10129 Torino, Italy; vincenzo.santucci@polito.it

**Keywords:** adsorption, circular economy, wastewater, refrigerator, WEEE

## Abstract

This study explored the performances of waste polyurethane foam (PUF) derived from the shredding of end-of-life refrigerators as an adsorbent for wastewater treatment. The waste PUF underwent a basic pre-treatment (e.g., sieving and washing) prior the adsorption tests. Three target pollutants were considered: methylene blue, phenol, and mercury. Adsorption batch tests were performed putting in contact waste PUF with aqueous solutions of the three pollutants at a solid/liquid ratio equal to 25 g/L. A commercial activated carbon (AC) was considered for comparison. The contact time necessary to reach the adsorption equilibrium was in the range of 60–140 min for waste PUF, while AC needed about 30 min. The results of the adsorption tests showed a better fit of the Freundlich isotherm model (R^2^ = 0.93 for all pollutants) compared to the Langmuir model. The adsorption capacity of waste PUF was limited for methylene blue and mercury (K_f_ = 0.02), and much lower for phenol (K_f_ = 0.001). The removal efficiency achieved by waste PUF was lower (phenol 12% and methylene blue and mercury 37–38%) compared to AC (64–99%). The preliminary results obtained in this study can support the application of additional pre-treatments aimed to overcome the adsorption limits of the waste PUF, and it could be applied for “rough-cut” wastewater treatment.

## 1. Introduction

According to the latest report published by the association of plastic manufacturers Plastic Europe [1], the demand for polyurethane in Europe was equal to 4 Mt in 2019, representing 7.9% of the total plastic demand. The main contributors to polyurethane requirement are the manufacturing of pillows and mattresses (31%), and the construction and building (24.5%), electrical and electronic (21.3%), and automotive (11%) sectors [1]. Of the 4 Mt/y polyurethane requested in Europe in 2019, approximately two thirds are in the form of foams (1.68 Mt flexible foam, 1 Mt rigid foam) [2]. In China, polyurethane output in 2011 reached 7.5 Mt, and polyurethane foam (PUF) accounted for 60% [3]. PUF wastes are product scraps, as the production of rigid polyurethane foam usually creates 15% of waste [3] and post-consumer waste materials. Of the total 29.1 Mt of plastic generated in Europe in 2019, approximately 1.5 Mt are made by PUF, of which one third is recycled, while the rest is incinerated or sent to landfill.

The scientific and technical literature offers several potential perspectives for material recovery from waste PUF, mostly as an oil absorbent [4,5,6,7], additive for construction materials [8,9,10,11], and adsorbent of pollutants from wastewater [12,13]. Nowadays, the market competition in the field of wastewater treatment technologies is increasing due to the need of achieving effective removal performances with limited costs. The most common adsorbent at the state-of-the-art level is activated carbon (AC), as dust or granular material, suitable for a variety of applications for drinking water, swimming pools, urban and industrial wastewater, etc. Alternatives to AC are oxides and zeolites, polymeric adsorbents (intended for application in industrial wastewater treatments, but their high costs of production and regeneration have prevented a broader application), and, developed more recently, low-cost adsorbents derived from wastes [14]. The literature is rich of studies that investigated the adsorption potential of industrial and agricultural wastes, particularly for the removal of dyes or metals from wastewater (Table 1) [15,16,17,18,19,20,21,22,23,24].

Table 1 provides an overview of the literature data describing the properties and performances of adsorbents deriving from different “parent” materials, categorized per type of contaminant. The most promising experimental applications of low-cost adsorbents were industrial wastewater containing dyes, metals, and halogenated compounds. The dose of adsorbent was in the range of 0.1–20.0 g/L, though it was higher for the removal of phenols. The specific surface area (SSA) directly affects adsorption, and high values are usually desirable to provide many adsorption sites. AC exhibited SSA values between 500 and 1500 m^2^/g [15,21]; however, adsorbents with relatively low values (<200 m^2^/g) could also achieve good adsorption capacities towards metals such as lead, cadmium, nickel, and cobalt [17]. The application of PUF as an adsorbent material for the removal of several pollutants from wastewaters is a recently investigated perspective [12,24]. PUF-based adsorbents achieved adsorption capacities between 20 and 30 mg/g for copper, cadmium, and chromium [13], making them less performant than commercial products, but still with a good adsorption capacity, higher than other waste-derived materials such as fly ash and hemp. Commercial AC is usually made from non-renewable resources or biomass transported over long distances, resulting in high environmental impacts due to feedstock and transportation, and in relevant energy demand [25]. The estimated impact on climate change of granular AC is 1.44 Kg CO_2_/kg adsorbent [20,26]. To sum up, a good adsorbent should: be made from a porous raw material with high SSA; have good affinity for the target contaminants; and have limited costs for raw material procurement-also including transportation, and for its preparation. To limit the environmental impacts, adsorbents with minimal energy consuming pre-treatments are preferable, and the feasibility of their regeneration after adsorption must be considered as well.

The interest of the scientific and industrial worlds is shifting towards waste-derived non-conventional adsorbents, derived from biological, agricultural, or industrial processes, which are available almost free of cost [27,28]. The porous structure of PUF is a desirable feature for an adsorbent because it provides numerous potential sites of adsorption; also, open-cell PUF can be successfully used in columns for the treatment of large volumes of wastewaters [14]. The potential as adsorbent of virgin PUF in combination with different reagents has been previously tested [29,30] but, to our knowledge, there are not many studies specifically exploring the application of “plain” (e.g., without modification of its chemistry) waste PUF as an adsorbent. The main goal of this study is to investigate the adsorption potential of waste PUF in the field of wastewater treatment technologies for the removal of inorganic and organic pollutants. Waste PUF is employed “as such” separated from end-of-life (EoL) refrigerators, after the application of minimal and simple physic treatments to eliminate the impurities (i.e., sieving and washing with water). The perspective explored by this study is coherent with the Circular Economy strategy of the actual European policy and regulations. This solution, if proven effective, can lead to a double potential benefit when costs and environmental burdens are reduced in comparison to the use of conventional adsorbents.

## 2. Materials and Methods

### 2.1. Waste PUF Origin and Characteristics

The tested material was waste PUF in a loose granular form derived from the shredding of EoL refrigerators (category 1 WEEE) at a TBD treatment plant managed by AMIAT in the metropolitan area of Turin, Italy. The waste PUF was sampled across 5 weeks (one sample per week) to account for any composition variability. The samples (1 kg each) were collected according to standard methods UNI 10802:2013 and UNI 14899:2006 at the end of the working day. The samples were assumed to be representative, considering that 3300 t/y EoL refrigerators entering the plant roughly correspond to over 300 items shredded per day [4]. The collected samples were quartered to obtain representative secondary samples for the characterization and adsorption tests. A complete characterization of the waste PUF is reported in a previous study [4], describing the investigation of the oil absorption potential of the same material (whole material and selected particle-size fractions). Compared to our previous study [4], this research explored the adsorption potential for wastewater treatment of the fraction of waste PUF with dimensions between 0.71 and 5 mm. The main features of the considered waste PUF are reported in Table 2. Commercial powdered Activated Carbon (AC) FILTERCARB RO, provided by Carbonitalia srl (Livorno, Italy) was chosen as reference material for the adsorption tests (Table 2).

### 2.2. Pre-Treatment

The waste PUF sampled in the WEEE shredding plant contained impurities such as plastic, paper, and metal. Before the adsorption tests, the waste PUF (fraction having dimensions between 0.71 and 5 mm) underwent a washing pre-treatment (0.125 L water/g PUF) aimed at removing the impurities as higher density (sink) fraction after 15 min of shaking at 150 rpm in an ARGOLAB SKI 4 orbital shaker. After a 3 min rest, the floating particles of PUF were collected and wet sieved at 0.71 mm with 0.03 L water/g PUF. The washed samples were drained for 10 days in ambient conditions (21 °C, relative humidity 63%) and stored in a dry container.

### 2.3. Target Pollutants

Three pollutants were considered in the adsorption tests: methylene blue, an organic compound present in paints used in textile and plastic industries; phenol, an organic pollutant derived from the polymer, chemical, and food industries; and mercury, a carcinogenic metal well known for its bioaccumulation potential in water reservoirs affected by industrial or mining activities [31]. The target pollutants solutions were prepared from the dilution in deionized water of: 1000 mg/L mercury solution Chem-Lab (Zedelgem, Belgium) 99.5+% phenol pellets Chem-Lab (Zedelgem, Belgium); 99.5% methylene blue (C_16_H_18_ClN_3_S · 3H_2_O) CarloErba Reagents (Cornaredo, MI, Italy).

The analyses of phenol and methylene blue were performed directly on the aqueous phases through an ONDA UV-30 SCAN UV-VIS spectrophotometer (at 269 and 668 nm, respectively). Mercury was analyzed through an NEX DE VS Rigaku XRF spectrometer.

### 2.4. Adsorption Tests

All adsorption experiments were performed in an ARGOLAB SKI 4 orbital shaker at 260 rpm and 20 °C. The AC was tested at a solid/liquid ratio equal to 0.75 g/L. All tests were conducted in three replicates.

Firstly, equilibrium tests were necessary to find the equilibrium time (t_eq_) for each target pollutant and the pre-treated waste PUF. Flasks of 250 mL were filled with 200 mL of 10 mg/L solution of each pollutant and 5 g of PUF (solid/liquid ratio equal to 25 g/L, chosen according to literature studies in Table 1). Three milliliter aliquots of solution were withdrawn after different time intervals, filtered on 0.45 µm cellulose ester syringe filters, and analyzed to measure the residual pollutant concentration. t_eq_ was determined as the time after which no decrease in the residual aqueous concentration was detected. q_eq_, i.e., the amount of pollutant adsorbed, was calculated as the difference between the initial concentration of pollutant in the liquid phase (C_Li_) and the residual value (C_Lf_).

The adsorption tests were performed in 50 mL falcon test tubes filled with 40 mL of pollutants solution and 1 g of pre-treated waste PUF (solid/liquid ratio equal to 25 g/L). The pollutant solutions were as follows: methylene blue: 0.5, 1, 2, 5, 7.5, 10, 15, 18, 20 mg/L; phenol: 6, 8, 10, 12, 14, 16, 19, 24, 30 mg/L; mercury: 2, 3, 4, 5, 6.5, 10, 12, 17, 22 mg/L. The tubes were shaken for an interval equal to the t_eq_ of each pollutant. The supernatant was separated from the solid phase through a Z20A Hermle centrifuge (Labortechnik GmbH, Wehingen, Germany) at 3500 rpm for 5 min, then filtered on 0.45 µm cellulose ester syringe filters and analyzed. The adsorption tests involved three replicates.

### 2.5. Isotherm Models

At a constant temperature, the process of adsorption can be described by an adsorption isotherm. After the equilibrium state has been reached, the concentrations of the adsorbate on the solid phase are plotted against concentrations of adsorbate in liquid phase. Two models were used for the interpretation of experimental data. The Freundlich model is based on Equation (1) [14]:

q_eq_ = K_f_ (C_eq_)^1/n^ (1)

where q_eq_ is the amount of adsorbate transferred on the sorbent at equilibrium; K_f_ is the capacity factor, a parameter that characterizes the strength of adsorption, and it is directly proportional to q_eq_. The exponent 1/n determines the curvature of the isotherm, and it denotes the intensity of adsorption.

The Langmuir model is based on Equation (2) [14]:

(2) $$q_{\mathrm{eq}}=\frac{q_{\max\;}b}{1+bC_{e}}\;\cdot {\;C}_{e}\;$$

where q_eq_ is the amount of adsorbate transferred on the sorbent at equilibrium; q_max_ is the maximum capacity of adsorption at saturation (assuming the formation of a single layer of adsorbed molecules); *b* is the Langmuir constant related to the adsorption energy.

## 3. Results and Discussion

### 3.1. Adsorption Equilibrium Tests

**Figure 1:**
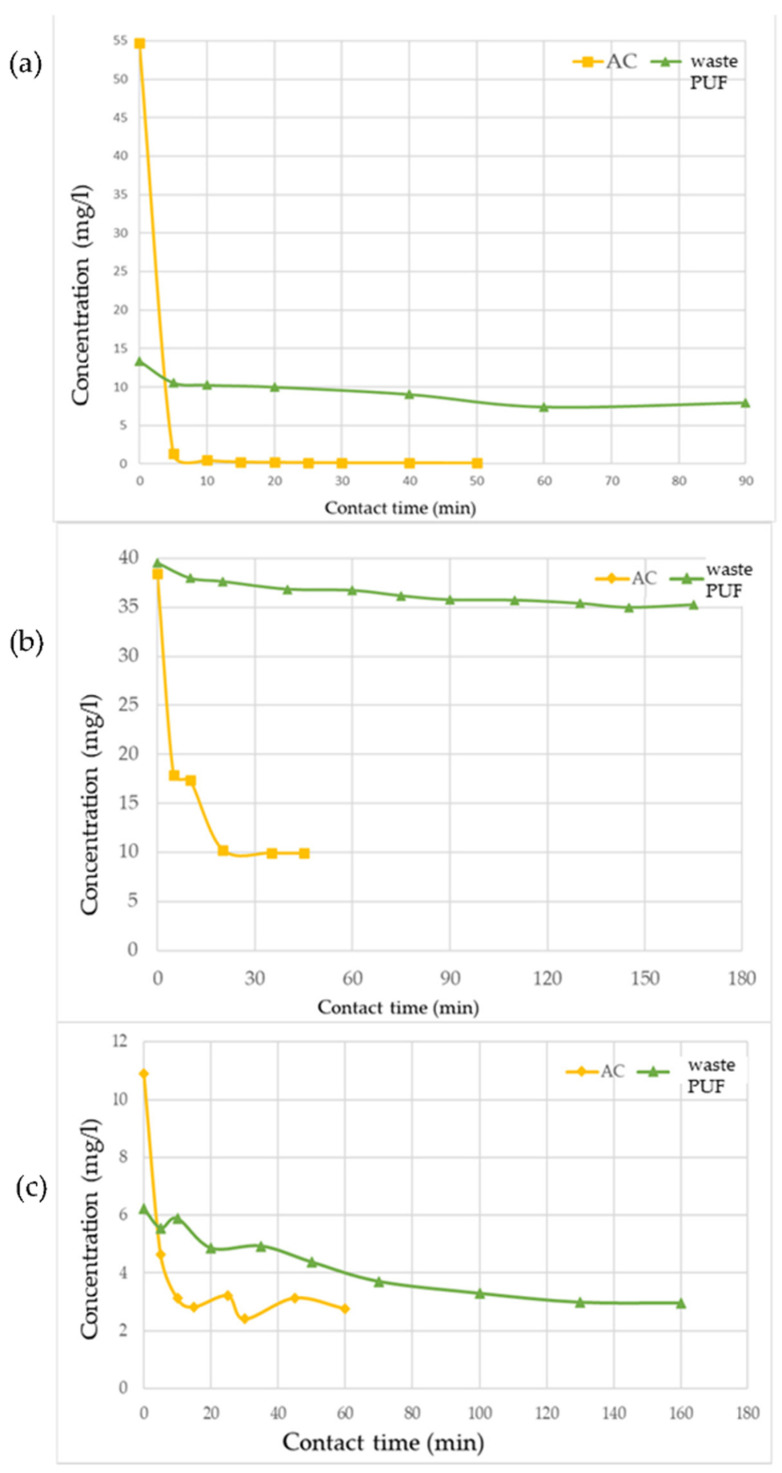
Results of the adsorption equilibrium tests performed on waste PUF and AC with (**a**) methylene blue, (**b**) phenol, and (**c**) mercury.

Considering the results of the equilibrium tests (Figure 1 and Table 3), for all target contaminants, the equilibrium of adsorption was reached more quickly with AC, so that the test was stopped earlier than for reactors with PUF, since no significant changes in liquid concentration were detectable. Compared to PUF, the much shorter t_eq_ found for AC is reasonably a consequence of its high specific surface area [14] and of the hydrophobic nature of polyurethane, which could make adsorption slower [32]. The pollutants reached the adsorption equilibrium on AC rather quickly (30–35 min), while waste PUF required much longer times: 60 min for methylene blue, and 135–140 min for phenol and mercury. From these preliminary tests and considering the amounts of pollutant transferred on the solid adsorbent (q_eq_), methylene blue exhibited the highest affinity, compared to phenol and mercury, both for waste PUF and AC (Table 3).

### 3.2. Adsorption Batch Tests

**Figure 2:**
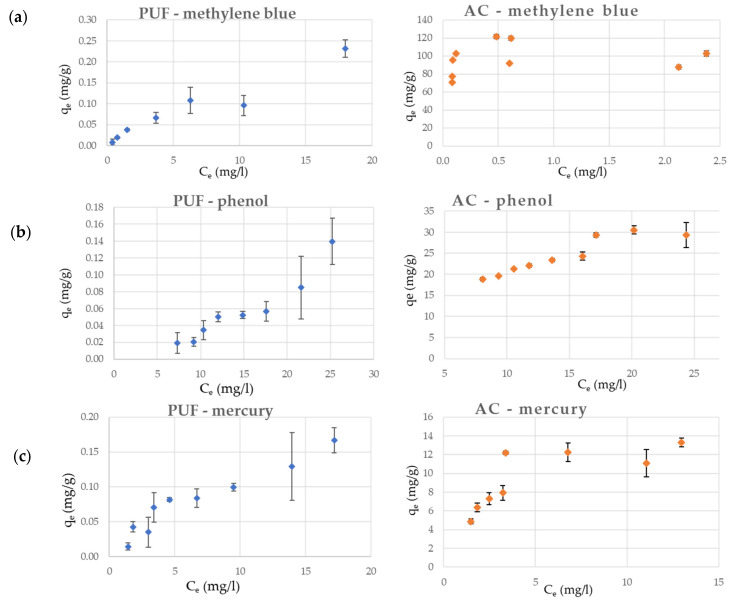
Results of the adsorption batch tests performed with waste PUF and AC in contact with (**a**) methylene blue, (**b**) phenol, and (**c**) mercury (C_e_: equilibrium concentration in the liquid phase; q_e_: equilibrium concentration in the solid phase).

The results of the adsorption batch tests (Figure 2 and Table 4) showed that the Freundlich isotherm model better fitted, compared to Langmuir model, the data related to waste PUF with an adequate correction factor (R^2^ = 0.93) for all the three pollutants. The adsorption capacity of waste PUF was moderate for methylene blue and mercury (K_f_ values around 0.02), while it was considerably lower for phenol (K_f_ around 1 × 10^−3^). Indeed, the maximum removal efficiency achieved from the batch tests by waste PUF (Table 5) was also rather limited: 12.2% for phenol and 37–38% for methylene blue and mercury.

The results of the adsorption tests performed on AC were described with higher accuracy by the Langmuir model for methylene blue (R^2^ = 0.99) and mercury (R^2^ = 0.95). Only in the case of phenol was the Freundlich model more adequate in describing the adsorption by AC (R^2^ = 0.88) than the Langmuir model (R^2^ = 0.59). The maximum removal efficiency achieved by AC for methylene blue was 99.9%, leading to very low residual concentrations in the liquid phase (C_Lf_ = 0.04 mg/L). The Freundlich model had inadequate experimental results obtained for methylene blue (R^2^ = 0.14), probably because when the concentrations at equilibrium are much lower than the initial concentrations, the adsorption is generally well described by a linear model. The Freundlich isotherm, which is in exponential form, cannot describe the linear range at very low concentrations. On the contrary, this limit case is well described by Langmuir model and when *b · C_Lf_ << 1*, it is equivalent to a linear isotherm. The higher q_max_ found for AC applied to the adsorption of methylene blue (135.13 mg/g), compared to q_max_ of phenol (26.11 mg/g) and mercury (0.05 mg/g), was realistically expected since the considered commercial AC is commonly applied for decolorization purposes.

Unfortunately, because of the different level of correction factors, a direct comparison of the two adsorbents was not possible. However, since the differences between the values of K_f_ and q_max_ obtained from waste PUF and AC were of several orders of magnitude almost in every case, it was evident that there was a considerable gap in favor of AC towards the adsorption of the considered target pollutants.

The results of this study were compared to literature data related to other novel and low-cost “non-conventional” (i.e., not commercial) materials tested for the adsorption of mercury (Table 6), phenol (Table 7) and methylene blue (Table 8). These materials, although at an experimental level, all underwent treatments aimed at improving their adsorption performances (e.g., activation for biomass-based sorbents, modification by addition of reagents for other materials). Literature data referred to mercury adsorption (Table 6) exhibited q_max_ in the range of 1.8–13 mg/g from the Langmuir model, and K_f_ between 0.02 and 19 L/mg from the Freundlich model, with correction factor values exceeding 0.9 for both isotherm models in all studies. Literature data on phenol adsorption (Table 7) found q_max_ values in the range of 38–285 mg/g from the Langmuir model, and K_f_ between 0.19 and 7.40 L/mg, with correction factor values exceeding 0.9 for both isotherm models in all studies. Methylene blue adsorption literature studies (Table 8) found typical values of q_max_ in the range of 29–2639 mg/g for the Langmuir model, and K_f_ between 0.82 and 1746 L/mg, with correction factor values around 0.8–0.9 for both isotherm models in all studies.

The fact that waste PUF did not show similar adsorption performances in the present study means that the tested material was not yet ready to provide competitive adsorption performances. Indeed, the gap was not so large when comparing the Freundlich parameters obtained from waste PUF (K_f_ = 0.019 L/mg) and other non-commercial adsorbents in contact with mercury solutions (K_f_ mostly in the range 0.02–4.50 L/mg, with one exception).

## 4. Conclusions

Investigating any possible opportunities for the recovery of plastics is a key step for supporting the European Circular Economy strategies. This research provides preliminary results about the adsorption properties of waste PUF deriving from the shredding of EoL refrigerators. In this study, waste PUF performances for the removal of methylene blue, phenol, and mercury from aqueous phases were compared with the ones of a commercial AC. Adsorption batch tests allowed to determine the adsorption isotherm parameters. The Freundlich isotherm model better fitted (R^2^ = 0.93), compared to the Langmuir model (R^2^ < 0.60), the adsorption of methylene blue, phenol, and mercury on waste PUF. In the considered experimental conditions, waste PUF showed a constrained affinity in adsorbing the target pollutants. The obtained Freundlich adsorption parameter K_f_ was around 0.02 L/mg for mercury and methylene blue, and 0.001 L/mg for phenol. These values were three or four orders of magnitude lower compared to commercial AC, and rather low when compared to the average adsorption capacities of non-commercial adsorbents according to the literature. Moreover, the long time required to reach the adsorption equilibrium (60–140 min depending on the pollutant) in the considered experimental conditions makes waste PUF direct application as an adsorbent rather challenging, especially in fixed-bed columns wherein short equilibrium times are desirable to design columns of reasonable height.

However, summarizing the results obtained in this study, it must be considered that waste PUF is a material deriving from a waste treatment process totally unintended for any adsorption application, and with a minimal preparation consisting only of sieving and washing. The results of this study can support the design of other pre-treatments aimed at overcoming the adsorption limits of the waste PUF “as such”. For instance, reducing the particle size of waste PUF, and thus increasing the available specific surface area, would benefit the rate of adsorption. After these additional studies, waste PUF could be applied for “rough-cut” wastewater treatment. When industrial wastewater with high pollution loads is delivered to treatment plants, a rough removal of contamination can be conducted with a relatively low-performant adsorbent such as PUF, prior to a second- more advanced purification process. Additionally, considering the comparison with the performances of other non-conventional (i.e., non-commercial) adsorbents, PUF exhibited the most promising affinity towards mercury. Therefore, further research could be conducted aiming at a feasible application of PUF for mercury removal.

## Figures and Tables

**Table 1:** Overview of studies describing the properties and performances of commercial and novel adsorbents towards different contaminants (SSA: specific surface area; C_Li_: initial concentration in the liquid phase; q_eq_: amount adsorbed on the solid phase; t_eq_: contact time).

Adsorbent Parent Material	SSA (m^2^/g)	Adsorbent Dose (g/L)	Contaminant	C_Li_ (mg/L)	q_eq_ (mg/g)	t_eq_	% Removal	Ref.
commercial activated carbon	698–1281	-	phenol	100–5000	200–270	1 h	99	[15]
biochars from lignocellulose biomass	63–211	-	phenol	100–5000	65–104	5 h	68	[15]
composite lignosulfonate sodium/cotton biochar	-	0.2	Pb	50–100	203.5	3 h	-	[16]
	-	0.2	methylene blue	5–30	109.1	24 h	-	
various bio-waste derived adsorbents	0.67–65.19	1–5	Cd	5–250	7.5–230.5	40–480 min	-	[17]
	-	0.6–15	Cr	5–8000	1.3–249	25–250 min	99.2	
	1.8–105	1–10	Pb	6.35–2000	8.6–909.1	30–300 min	>94	
	0.853–450	0.4–10	Cu	5–100	2.1–19.5	30–360 min	-	
	0.75–17.38	1–5	Ni	23–250	0.3–285.7	20–180 min	-	
	0.75–206.8	1–10	As	2.5–500	0.42–133	60–360 min	-	
	59–450	1–18	Zn	20–5000	2.4–68.5	20–300 min	-	
	0.78–186	0.6–4	Co	10–600	14.8–349.6	3–120 min	-	
maize straw ash	38.3	0.2–1.2	perfluorinated compounds	1–500	811	48 h		[18]
chitosan-based polymer	-	-	perfluorinated compounds	20–550	1452	32 h	40–60	[19]
non-ionic resins	-	-	perfluorinated compounds	0.01–5	37–46	10–96 h	-	[20]
industrial by-products (blast furnace residues, fly ash, red mud)	4.5–1740	0.25–8	different commercial dyes	-	1.3–390	2–72 h	-	[21]
	3–1440	0.1–50	Cu, Zn, Cr, As, Ni, Cd, Pb	1–4000	1–140	3–72 h	-	
	69–380	0.2–200	phenols	200–1500	11.4–190.2	2–8 h	-	
physically immobilized PUF	-	4	Cr	10	-	2 h	98.6	[22]
thiazolidinone steroids impregnated PUF	-	1	Cd	5–10	-	1 h	94–96	[23]
candle sooth PUF	-	50	Rhodamine B	50	15.066	150 min	96	[24]

**Table 2:** Main features of the considered waste PUF and of the reference commercial AC.

Parameter	Measure Unit	Waste PUF	AC
Specific Surface Area	m^2^/g	-	>1750
ash at 550 °C	%	10.40 ± 1.60	<3.00
bulk density	kg/m^3^	47.57	<350.00
pH in water	pH units	8.02 ± 0.16	5.00 ± 1.00
moisture	%	<0.1	<10.0
particle size distribution	mm	0.710 ÷ 5.000	0.015 ÷ 0.110
electrical conductivity	µS/cm	125.50 ± 12.70	<200.00

**Table 3:** Details and results of the adsorption equilibrium tests performed on PUF and AC (C_Li_: initial concentration in the liquid phase; C_Lf_: final concentration in the liquid phase; t_eq_: equilibrium time; q_eq_: amount of contaminant transferred on the sorbent).

Adsorbent	Adsorbate	Adsorbent Dose (g/L)	C_Li_ (mg/L)	C_Lf_ (mg/L)	t_eq_ (min)	q_eq_ (mg/kg)
Waste PUF	methylene blue	25.00	12.50	7.49	60	0.24
	phenol	25.00	40.00	35.00	140	0.17
	mercury	25.00	6.00	2.97	135	0.13
AC	methylene blue	0.75	55.73	0.14	30	74.11
	phenol	0.75	38.48	9.89	30	38.12
	mercury	0.75	10.90	2.41	35	11.32

**Table 4:** Values of Freundlich (K_f_, n) and Langmuir (q_max_, b) isotherm models’ parameters resulting from the interpolation of the experimental data derived from batch adsorption tests with waste PUF and AC.

Pollutant	Adsorption Model	Waste PUF			AC		
	Freundlich	K_f_ (L/mg)	1/n	R^2^	K_f_ (L/mg)	1/n	R^2^
methylene blue		0.022	0.797	0.93	101.110	0.056	0.14
phenol		0.001	1.517	0.93	7.020	0.468	0.88
mercury		0.019	0.784	0.93	5.170	0.39	0.68
	Langmuir	q_max_ (mg/g)	b (L/mg)	R^2^	q_max_ (mg/g)	b (L/mg)	R^2^
methylene blue		0.363	0.061	0.54	135.130	1.480	0.99
phenol		0.098	0.023	0.57	26.110	0.085	0.59
mercury		0.349	0.048	0.43	0.059	0.410	0.95

**Table 5:** Maximum removal efficiencies achieved in batch adsorption tests performed with waste PUF and AC (C_Li_: initial concentration in the liquid phase; C_Lf_: final concentration in the liquid phase).

Pollutant	C_Li_ (mg/L)		C_Lf_ (mg/L)		% Removal	
	Waste PUF	AC	Waste PUF	AC	Waste PUF	AC
methylene blue	1.27	48.32	0.78	0.04	38.50	99.90
phenol	28.73	22.19	25.24	8.05	12.20	63.70
mercury	2.87	12.53	1.81	3.38	37.00	73.00

**Table 6:** Performances of some non-commercial adsorbents tested for the removal of mercury.

	Langmuir Model			Freundlich Model			Temperature	Ref.
Adsorbent	q_max_ (mg/g)	b (L/mg)	R^2^	K_f_ (L/mg)	n	R^2^	°C	
biochar	6.54	0.328	0.995	1.72	2.204	0.987	25	[33]
modified biochar	9.15	0.608	0.992	3.22	1.803	0.949	25	[33]
bentonite	2.01	0.125	0.984	0.29	2.505	0.995	25	[33]
biochar-bentonite composite	11.72	0.749	0.991	4.50	2.482	0.981	25	[33]
hydrated lime	12.93	0.070	0.990	0.02	50	1.00	room	[34]
co-doped molybdenum selenide (nitrogen and sulfur)	-	-	-	18.96	0.40	0.988–0.995	25	[35]
chitosan modified PUF	1.84	0.989	0.888	0.30	0.623	0.942	room	[36]

**Table 7:** Performances of some non-commercial adsorbents tested for the removal of phenol.

	Langmuir Model			Freundlich Model			Temperature	Ref.
Adsorbent	q_max_ (mg/g)	b (L/mg)	R^2^ ^-^	K_f_ (L/mg)	n	R^2^	°C	
zeolite/AC composite	37.92–40.31	0.022–0.032	0.929–0.944	5.74–7.40	0.20–0.32	0.998	25–40	[37]
modified halloysite nanotubes	-	-	-	0.19	0.99	0.987	25	[38]
biochar from lignocellulose biomass	65.00–104.00	0.00054–0.00094	-	1.10–4.80	0.29–0.52	-	25	[39]
Biochar from sewage sludge	216.76	0.0067	0.998	2.66	0.7635	0.987	35	[40]
carbon pellets from cigarette butts	211.45–285.11	0.0096–0.015	0.976	-	-	-	10–40	[41]

**Table 8:** Performances of some non-commercial adsorbents tested for the removal of methylene blue.

	Langmuir Model			Freundlich Model			Temperature	Ref.
Adsorbent	q_max_ (mg/g)	b (L/mg)	R^2^	K_f_ (L/mg)	n	R^2^	°C	
biochar from soybean	2488.00–2639.00	0.39–1.04	0.999–1.00	1672.00–1746.00	11.65–16.95	0.849–0.912	25	[42]
graphene-oxide-based nanocomposites from rice husks	478.47–632.91	3.66–10.38	0.859–0.985	334.37–422.22	6.18–6.83	0.893–0.929	ambient	[43]
corn husk powder	30.30	0.003	0.949	8.51	2.27	0.827	25–28	[44]
biochar from eucalyptus	114.60	20.68	0.901	86.58	0.085	0.980	30	[45]
zeolite clays combined with ZnTiO_3_/TiO_2_	29.14–49.81	0.43–1.00	0.990	11.98–18.80	0.30–0.38	0.970	ambient	[46]
adsorbent based on magnetic metal−organic compounds	148.80	0.051	0.961	17.40	0.47	0.992	ambient	[47]
biochar from Paulownia wood	255.89	0.003	0.886	0.82	40.27	0.839	20–40	[47]

## Data Availability

The data presented in this study are available on reasonable request from the corresponding author.
